# Engagement With Motivational Interviewing and Cognitive Behavioral Therapy Components of a Web-Based Alcohol Intervention, Elicitation of Change Talk and Sustain Talk, and Impact on Drinking Outcomes: Secondary Data Analysis

**DOI:** 10.2196/17285

**Published:** 2020-09-01

**Authors:** Ajla Mujcic, Stuart Linke, Fiona Hamilton, Alexandria Phillips, Zarnie Khadjesari

**Affiliations:** 1 Erasmus School of Social and Behavioural Sciences Erasmus University Rotterdam Rotterdam Netherlands; 2 Trimbos Institute The Netherlands National Institute of Mental Health and Addiction Utrecht Netherlands; 3 Camden and Islington Mental Health Trust London United Kingdom; 4 eHealth Unit Research Department of Primary Care and Population Health University College London London United Kingdom; 5 Department of Psychology King's College London London United Kingdom; 6 School of Health Sciences University of East Anglia Norwich Research Park Norwich United Kingdom

**Keywords:** eHealth, digital health, self-management, alcohol use, motivational interviewing, cognitive behavioural therapy, engagement

## Abstract

**Background:**

Down Your Drink (DYD) is a widely used unguided web-based alcohol moderation program for the general public based on cognitive behavioral therapy (CBT) and motivational interviewing (MI); it provides users with many opportunities to enter free-text responses.

**Objective:**

The aim of this study was to assess participants’ use of key CBT and MI components, the presence of change talk and sustain talk within their responses, and whether these data are associated with drinking outcomes after 3 months.

**Methods:**

An exploratory secondary data analysis was conducted on data collected in 2008 from the definitive randomized trial of DYD (N=503). Past week alcohol use at baseline and 3-month follow-up were measured with the TOT-AL. Covariates included baseline alcohol use, age, gender, education level, and word count of the responses. Use of MI and CBT components and presence of change talk and sustain talk were coded by two independent coders (Cohen κ range 0.91-1). Linear model regressions on the subsample of active users (n=410) are presented along with a negative binomial regression.

**Results:**

The most commonly used component was the listing of pros and cons of drinking. The number of listed high-risk situations was associated with lower alcohol use at 3-month follow-up (B_adj_ −2.15, 95% CI −3.92 to −0.38, *P*=.02). Findings on the effects of the percentage of change talk and the number of listed strategies to deal with high-risk situations were inconsistent.

**Conclusions:**

An unguided web-based alcohol moderation program can elicit change talk and sustain talk. This secondary analysis suggests that the number of listed high-risk situations can predict alcohol use at 3-month follow-up. Other components show inconsistent findings and should be studied further.

## Introduction

Interventions aimed at reducing risky alcohol use are diverse and vary in many ways, including their mode of delivery (eg, in-person, bibliotherapy, digital), theoretical approach, and length (ranging from ultrabrief to extended interventions). This variation is also reflected in digital alcohol interventions [1]. Ultrabrief digital alcohol interventions are usually limited to self-monitoring exercises and personalized feedback (eg, decisional balance feedback [2]). Brief interventions can consist of self-monitoring exercises combined with personalized feedback and additional modules such as identification of high-risk situations, which help reduce alcohol use in specific situations (eg, the web-based personalized feedback program Drinktest [3]). On the other hand, extended interventions offer a digital form of intensive treatment, including multiple sessions (eg, the self-help alcohol intervention Balance [4]).

Several systematic reviews have shown that web-based alcohol interventions can be effective at reducing alcohol use in adult populations, finding small and moderate effect sizes [5-7]. Moderators of effectiveness have been studied and include length of intervention [1], level of guidance, setting, and integrated therapeutic principles [5]. Multiple studies have examined ways to increase engagement with alcohol and other health behavior interventions. Although these studies have conceptualized engagement in different ways, such as the received dose, adherence, degree of involvement over a longer period of time, or process of linked behaviors [8], engagement is mostly linked to frequency and length of use or to the use of specific components. However, few studies have examined users’ engagement with specific components of web-based alcohol interventions in detail [1].

Furthermore, although many alcohol interventions are partly based on motivational interviewing (MI) [1], few studies have explored the presence of change talk and sustain talk, which are important components of this “collaborative, goal-oriented style of communication with particular attention to the language of change, designed to strengthen personal motivation for and commitment to a specific goal by eliciting and exploring the person’s own reasons for change within an atmosphere of acceptance and compassion [9].” Change talk can be defined as language referring to movement toward change of a target behavior, including verbalizations of consideration, motivation, or commitment to change. Sustain talk is language referring to movement toward sustaining the target behavior and the status quo. In a meta-analysis from 2018 on MI processes, including 21 studies on alcohol use that mostly involved face-to-face treatment, it was found that a higher proportion of change talk was associated with reductions in risk behaviors [10]. One recent study looked at the presence of change talk and sustain talk in a web-based intervention; however, the intervention targeted physical activity, not alcohol use [11]. To the best of our knowledge, no study has explored the presence of change talk and sustain talk in a web-based alcohol intervention.

The Down Your Drink (DYD) website is a web-based alcohol intervention aimed at the general population [12]. DYD has some distinct features. First, it is targeted at opportunistic electronic help (e-help)–seekers who are not enrolled in an alcohol treatment pathway and who differ from dependent help seekers [13]. Another important feature of DYD is that it is one of the first web-based extended alcohol interventions based on MI techniques and cognitive behavioral therapy (CBT). Other examples are web-based self-help alcohol interventions from the Netherlands [14] and Norway [4]. DYD attempted to translate components of usual face-to-face treatment to a web-based, unguided setting and encourages self-monitoring of drinking behavior in the Drinking Episode Diary. Lastly, it offers many opportunities for free-text responses, which provides an opportunity to study the way web-based alcohol interventions are understood and used.

The original trial reported descriptive data on engagement with DYD, namely number of logins and number of pages viewed [15]. However, user data, that is, actual responses provided by participants to MI questions designed to strengthen personal motivation for and commitment to a specific goal, were not analyzed. Understanding how DYD was used and whether it encouraged people to think about changing their drinking is imperative to optimize future web-based alcohol programs. With this secondary analysis, we aim to answer the following research questions:

1. Does DYD elicit change talk and sustain talk?
2. Are the MI and CBT components of DYD used actively (ie, users responded to questions related to the component at least once)?
3. Do i) user responses indicating change talk and sustain talk and ii) users making use of the separate MI and CBT components have an impact on change in alcohol consumption (intervention effectiveness)?

## Methods

### Design

This paper reports on an exploratory secondary data analysis conducted on data from the definitive randomized trial of DYD (see the next section for further details). Ethical approval for the secondary analyses conducted in this study was granted by the University College London (UCL**)** Research Ethics Committee (“Engagement with internet-based alcohol moderation intervention ‘Down Your Drink’” project ID: 3770/002).

### DYD Trial

Data used for this study were collected during the definitive DYD trial from October 2007 to May 2009. This two-arm randomized controlled trial aimed to assess the effectiveness of DYD in reducing alcohol consumption and alcohol-related harm at 3- and 12-month follow-up. People aged 18 years or older who had internet access, scored 5 or more on the Alcohol Use Disorders Identification Test-Consumption (AUDIT-C), understood written English, and were willing to complete follow-up questionnaires could participate. A total of 2652 adults with scores of 5 or higher on the AUDIT-C were included [16]. During the pilot and main trial extension phases, another 5238 participants were included. The primary outcome measure of alcohol consumption in the past week was collected using the TOT-AL [17]. The entire study was conducted on the internet. A more extensive description of the main trial procedures can be found in Murray et al [18] and Wallace et al [15].

### Participants

For this study, we included the DYD trial participants who were randomized to the intervention group in 2008 (the only full calendar year of inclusion during the main trial) and who reported their alcohol use at 3-month follow-up. This ensured that participants who enrolled at various time periods during the year were all included. The sample provided a sufficient yet manageable number of participants for the analysis. In 2008, a total of 2543 participants were randomized; of those, 1271 were randomized to the intervention group. After removing participants who withdrew consent during the course of the study (n=41) and those who had not reported their alcohol use at 3-month follow-up (n=727), the final sample consisted of 503 participants. In addition to this full sample, we independently analyzed the active use sample, a subsample of 410 participants who actively engaged with the program at least once, defined as those who responded to at least one of the questions within the DYD program.

### Intervention

DYD is a web-based alcohol intervention that is primarily based on two evidence-based approaches that are widely used in the psychological treatment of alcohol misuse, namely MI and CBT; the latter is a goal-oriented therapeutic approach that systematically addresses dysfunctional emotions, behaviors, and thought processes. For example, CBT includes components urging participants to set a goal, recognize high-risk situations, and articulate their attitudes concerning (moderating) their drinking behavior. Self-monitoring of drinking behavior (eg, amount, type, setting, cost) is facilitated in the Drinking Episode Diary. The Alcohol Units Counter is another self-monitoring exercise; however, it does not keep track of changes in drinking over time. DYD delivers the MI approach by presenting a series of questions prompting free-text responses. The effectiveness of MI depends on how people respond to the questions, that is, whether their responses suggest that they wish to reduce their drinking (ie, change talk) or not (ie, sustain talk). For a more extensive description of the intervention, see [12].

### Measures

Measures from the DYD trial included in the current analyses are past week self-reported alcohol consumption in UK units (ie, 1 unit=8 grams of ethanol) at baseline and 3-month follow-up measured using the TOT-AL (total past week alcohol consumption) [17], age (continuous variable), gender (male/female), and education level (university degree or equivalent/A Levels or equivalent)/General Certificate of Secondary Education (GCSE) or equivalent/other qualifications/no qualifications). Ethnicity and relationship status were used to describe the sample but were not included as covariates in the model. This study included measures on the use of DYD components, which will be described further in the Qualitative Analysis section. To assess the effects of change talk and sustain talk, the percentage of change talk (change talk frequency over the sum of change talk frequency plus the sustain talk frequency) was included as recommended [19]. Lastly, the total number of words entered into the program was computed for each participant.

### Qualitative Analysis

The coding scheme (see Multimedia Appendix 1) included the main MI components and CBT components, and it was informed by the Client Language EAsy Rating (CLEAR) coding system [19] and items from the Revised Cognitive Therapy Scale (CTS-R) [20]. CLEAR is a coding system that can be used to assess change talk and counter change talk (ie, sustain talk) in a participant’s responses. It was originally developed for in-therapy client language. MI components that were coded for included the presence of change talk, the presence of sustain talk, listing of the pros and cons of drinking alcohol, and noting of values (what is most important and meaningful to oneself). For each of these components, participants were assigned either a 1 (present) or a 0 (not present) depending on their responses. Furthermore, we coded the number of times the following were uttered: change talk, sustain talk, and pros and cons of drinking. Note that the frequency of change talk included the uttered cons of drinking and all other change talk present in response to the other questions as evaluated using CLEAR. The frequency of sustain talk included the number of mentioned pros of drinking and all other sustain talk present in response to other questions within the program.

The CTS-R is a scale for measuring therapist competence in cognitive therapy; it lists several key components of cognitive therapy, which helped us identify the main CBT components. We coded the following CBT components: setting a start date for alcohol moderation, setting a goal for alcohol moderation, completing another part of the moderation plan (eg, noting someone who might help), noting alcohol use prior to DYD, noting high-risk situations for alcohol use (eg, places, people, emotions), noting strategies to deal with high-risk situations, exploring feelings of craving, exploring relapse prevention (eg, thinking back to circumstances around a previous relapse), making a relapse plan (eg, stating who to call in case of a lapse), exploring thoughts (about drinking), monitoring alcohol use (ie, type, frequency, and amount of drinking). For all these codes, participants were assigned either a 1 (present) or a 0 (not present) depending on their responses. Furthermore, we coded the number of times the following were mentioned: high-risk situations and strategies to handle high-risk situations.

The coding was completed by two coders (AM and AP); SL was consulted on any discrepancies. Each coder coded 50% of the sample independently, and 51 (10%) of the sample were coded by both. Interrater reliability was high for both the dichotomous variables, as addressed with Cohen κ (range 0.91-1, mean 0.97), and the continuous variables, as addressed with intraclass correlation (range 0.90-0.99, mean 0.97). Continuous variables included the number of times the following were present: change talk, sustain talk, pros, cons, high-risk situations, and ways to handle high-risk situations.

### Quantitative Analysis

Descriptive statistics for each DYD use variable were computed to assess the presence of change talk and sustain talk within the responses and the use of CBT and MI components. All analyses described in this study are exploratory, as they were not preregistered. Multiple hierarchical regression models and analyses were used to assess the predictive value of the different components as well as of the change talk and sustain talk on alcohol use reduction at 3-month follow-up. In accordance with CLEAR guidelines, change talk and sustain talk were entered into the model as percentage change talk [19]. In the first step, a linear model was generated containing the baseline drinking level and the following covariates: number of words entered in all responses by the participant, age, gender, and education level. This model was then compared to a linear model containing all the variables of interest to assess the added explained variance of the full model. The distribution of the alcohol consumption outcome was highly skewed; therefore, two sensitivity analyses were conducted. The first sensitivity analysis used a linear model in which the alcohol consumption variables were log-transformed (after adding 1 unit per week). In the second sensitivity analysis, a negative binomial regression was performed, as the log transformation of drinking outcomes did not result in perfectly normally distributed data. A negative binomial regression as recommended by Atkins et al [21] was used to model the alcohol count data. Model estimates are presented for data from the subset of users who engaged with the program at least once (ie, the active use sample). All analyses were conducted in R version 3.5.1 (R Project) [22].

## Results

### Sample Characteristics

The majority of the sample was female (308/503, 61.2%) and aged 18 to 73 years (median 41, mean 40, SD 11.24) with a predominant ethnicity of White British (419/503, 83.3%). Within the total sample, 93 participants had registered accounts but did not engage with any of the interactive elements of the web-based program, creating a subgroup (410/503, 81.5%) who responded to at least one question. Baseline alcohol consumption in the past 7 days was 54 units on average (median 45.4, range 0-322.1, SD 37.6) in the total sample, and 55.5 units (median 47.8, range 0-322.1, SD 36.0) in the active use sample. Past week alcohol consumption at 3-month follow-up was 37.2 units on average (median 30.6, range 0-284.6, SD 31.2) in the total sample and 37.4 units (median 30.2, range 0-284.6, SD 31.4) in the subsample. The characteristics of both the total sample and the active use sample are displayed in Table 1; there were no notable differences.

**Table 1:** Demographic characteristics of the participant sample (N=503).

Baseline characteristic		Full sample (N=503), n (%)^a^	Active use sample^b^ (n=410), n (%)^a^
**Age (years)**			
	18-34	165 (32.9)	125 (30.6)
	35-44	150 (29.9)	121 (29.6)
	45-54	138 (27.5)	123 (30.1)
	55-73	49 (9.8)	40 (9.8)
	Not specified	1 (0.2)	1 (0.2)
**Gender**			
	Female	308 (61.2)	258 (62.8)
**Education level**			
	University degree or equivalent	289 (57.4)	245 (59.6)
	A Levels or equivalent	88 (17.5)	68 (16.5)
	GCSE^c^ or equivalent	73 (14.5)	56 (13.6)
	Other qualifications	39 (7.8)	32 (7.8)
	No qualifications	14 (2.8)	10 (2.4)
**Relationship status**			
	Married/long term relationship	316 (62.8)	258 (62.8)
	Unmarried/divorced	187 (37.2)	152 (37.1)
**Ethnicity**			
	White British	419 (83.3)	344 (83.9)
	White other	46 (9.1)	36 (8.8)
	White Irish	25 (5.0)	17 (4.1)
	Mixed	4 (0.8)	4 (1.0)
	Asian/Asian British	3 (0.6)	3 (0.7)
	Black African/Black Caribbean/Black British	2 (0.4)	2 (0.5)
	Other	4 (0.8)	4 (1.0)

^a^Due to rounding, percentages may not total 100.

^b^Subsample of participants who responded to at least one question in the Down Your Drink web-based alcohol intervention.

^c^GCSE: General Certificate of Secondary Education.

### Change Talk and Sustain Talk

On average, participants entered almost three times the number of segments classified as change talk (mean 12.3, SD 9.79) than as sustain talk (mean 4.1, SD 2.00) (see Table 2). The percentage of change talk was 61.5% on average (median 70%, range 0% to 100%). The majority of participants had entered at least one segment that was classified as change talk (341/410, 83.2%). Change talk encompassed the mention of benefits of quitting/moderating drinking, mention of disadvantages of current drinking behavior, recognition of problematic drinking behavior or needing help, and explicating the intent to change drinking behavior. Noting other people’s desire for the participant to quit drinking was not classified as change talk. Examples:

I want to be in control of everything I do instead of putting things off because I want to drink alcohol.

I need help to stop drinking.

I know I am drinking too much and this number of units does not surprise me.

Sustain talk encompassed naming benefits of current drinking behavior, mentioning perceived disadvantages or fears about quitting/moderating drinking, and expressing a lack of self-confidence in changing current drinking behavior. Examples:

I feel positive about life and make plans when I have been drinking.

I look forward to drinking.

[…] But fear [losing] the good side of alcohol and how it makes me feel.

Don’t feel confident to change.

I have no will power.

### Use of MI and CBT Components

Descriptive information on the use of the MI and CBT components is shown in Table 2. Listing the pros or cons of drinking was the component actively engaged with by the most participants (337/410, 82.2%). The components engaged with by the fewest participants were exploring cravings (16/410, 3.9%), exploring relapse prevention (24/410, 5.9%), and making a relapse plan (9/410, 2.2%).

**Table 2:** Use of motivational interviewing and cognitive behavioral therapy components by participants in the Down Your Drink web-based alcohol intervention.

Component		Active use sample (n=410)				Users who engaged in component at least once^a^	
		n (%)	Mean (SD)	Median	Range	Mean (SD)	Median
**MI^b^ components (n=352, 85.9%)**							
	Change talk^c^	341 (83.2)	10.3 (10.02)	8	0-90	12.3 (9.79)	9
	Sustain talk^c^	329 (80.2)	3.3 (2.43)	3	0-13	4.1 (2.00)	4
	Percentage change talk^d^	N/A^e^	61.5% (N/A)	70%	0%-100%	72.5% (N/A)	73.7%
	Any listed pros or cons	337 (82.2)	N/A	N/A	N/A	N/A	N/A
	Pros^c^	334 (81.5)	2.9 (1.98)	3	0-12	3.6 (1.49)	4
	Cons^c^	327 (79.8)	6.8 (4.87)	7	0-24	8.4 (3.99)	7
	What is important	311 (75.9)	N/A	N/A	N/A	N/A	N/A
**CBT^f^ components (n=288, 70.2%)**							
	Setting start date	132 (32.2)	N/A	N/A	N/A	N/A	N/A
	Setting a drinking goal	129 (31.5)	N/A	N/A	N/A	N/A	N/A
	Completing another part of moderation plan	136 (33.2)	N/A	N/A	N/A	N/A	N/A
	Noting alcohol use before DYD	251 (61.1)	N/A	N/A	N/A	N/A	N/A
	Noting high-risk situations^c^	113 (27.6)	1.1 (3.04)	0	0-21	4.1 (4.60)	2
	Noting strategies^c^	103 (25.1)	2.7 (7.11)	0	0-48	10.9 (10.67)	7
	Exploring cravings	16 (3.9)	N/A	N/A	N/A	N/A	N/A
	Exploring relapse prevention	24 (5.9)	N/A	N/A	N/A	N/A	N/A
	Making a relapse plan	9 (2.2)	N/A	N/A	N/A	N/A	N/A
	Exploring thoughts about drinking	49 (12.0)	N/A	N/A	N/A	N/A	N/A
	Monitoring drinking^c^	141 (34.4)	12.0 (47.30)	0	0-654	34.8 (75.73)	9

^a^Responses by subset of participants who entered at least one response to a question corresponding with the component (eg, for high-risk situations, a subset of 113 participants entered at least one high-risk situation).

^b^MI: motivational interviewing.

^c^Mean and median refer to the number of statements corresponding to the component.

^d^Mean, median, and range are presented for percentage change talk.

^e^N/A: not applicable.

^f^CBT: cognitive behavioral therapy.

#### MI Components

Participants responded to questions about the pros and cons of drinking by listing their own perceived pros and cons of drinking. Pros often referred to the experience of alcohol drinking as enjoyable; they also stated that it aided their relaxation and confidence in social situations. Examples:

It makes me more confident and talkative.

It takes the edge off things.

I enjoy the taste.

Cons of drinking most often centered on worries about alcohol drinking affecting the participants’ health condition, lowered mood after alcohol drinking, and worries about drinking removing inhibitions, which sometimes led to regrettable behavior. Other cons focused on more practical issues, such as the cost of drinking. Examples:

It’s bloody expensive!

Feeling low and depressed the next day and having no motivation

Concerns about health effects

Out of control

Say/do things I regret

A specific part of the DYD program encouraged participants to think about what is most important to them. The responses differed; most participants gave to-the-point answers, whereas others elaborated extensively on the role alcohol played in their lives. Examples:

Good health, friends, my family.

The most important things in my life are my children.

My job and how drinking affect that

My children! […] I want to be a good role model for them and never make them feel worried about me when they are older. I want to be able to have just one drink and not feel as though I can't stop. I'd like to enjoy alcohol socially without feeling ashamed of myself the next day. I want to lose weight, feel good about myself every day and be as healthy as possible for my children and future grandchildren.

…

#### CBT Components

Some CBT components were rarely engaged with (eg, making a relapse plan and exploring cravings); however, those that were used showed a large variation in responses, with some participants adding large amounts of detail to their responses and others noting only keywords. Most responses were related to alcohol drinking, but not all; for example, in the “goal setting” component, some participants related a broader life goal instead of a specific drinking goal. Examples of goal setting nonspecific to drinking are:

To repay my debts and be good at my job

To keep friends with people, to make new friends and to be respected

Lose weight, be better Mum, get pregnant

Drinking goals also varied in their specificity. Some participants set a clear maximum number of drinks, and others stated a general goal of drinking less. Examples:

To reduce my drinking and the habits that surround it.

To put a stop to the binge drinking sessions

Limit myself to 2 large glasses in the evening and have x2 alcohol free nights

Stated high-risk situations varied from negative feelings to times of day, social situations, and events. Peer pressure was also frequently mentioned. Responses showed an overall good comprehension of the questions, although some answers were unspecific. Examples:

Anger, loneliness, despair

Boredom in the evenings

Going on holiday at New Year will also be a time of temptation

Seeing friends. Alcohol just makes the conversation flow easier. This is probably the hardest situation.

Getting another [because] everyone else is

Social situation

Strategies to cope with high-risk situations could either be selected from a list or noted in free-text responses. Free-text responses were generally unspecific, aimed at distraction or avoidance, and did not account for any difficulties that might arise from the coping strategies. However, some responses seemed to have incorporated strategies that were suggested in the DYD program. Examples:

Don't buy it

Read or watch a film

Doing activities

Drinking slowly and make glass last longer

### Use of DYD and Drinking Outcomes

Model estimates are presented for data from the subset of users who engaged with the program at least once, namely the active use sample (n=410). Lower alcohol use at 3 months, when controlled for age, gender, education level, alcohol use at baseline, and number of words, was associated with a greater percentage of change talk (B_adj_ −0.17, 95% CI −0.32 to −0.02, *P*=.03) and a higher number of listed high-risk situations (B_adj_ −2.15, 95% CI −3.92 to −0.38, *P*=.02). In the unadjusted models, listing any high-risk situations (B_unadj_ −8.31, 95% CI −14.88 to −1.74, *P*=.01) and the number of listed strategies to deal with high-risk situations (B_unadj_ −8.31, 95% CI −14.88 to −1.74, *P*=.01) also showed significant associations with alcohol use at 3-month follow-up but not when adjusted for all other components (adjusted R^2^ 0.38). The complete results are shown in Table 3.

**Table 3:** Linear model estimates for the subsample of active users (n=410).

Model and variable			Unadjusted^a^				Adjusted^b^		
			B	95% CI	*P* value	B		95% CI	*P* value
**Null model^c^**									
	**Covariates**								
		Baseline alcohol use	N/A^d^	N/A	N/A	0.54		0.47 to 0.62	<.001^e^
		Gender (male)	N/A	N/A	N/A	−6.01		−11.45 to −0.56	.03^e^
		Education (A level)	N/A	N/A	N/A	4.25		−2.75 to 11.24	.23
		Education (O level)	N/A	N/A	N/A	4.52		−2.94 to 11.99	.23
		Education (other)	N/A	N/A	N/A	2.22		−7.34 to 11.79	.65
		Education (no qualification)	N/A	N/A	N/A	−4.70		−20.70 to 11.30	.56
		Age	N/A	N/A	N/A	0.27		0.03 to 0.51	.03^e^
		Number of words	N/A	N/A	N/A	0.00		−0.01 to 0.02	.53
**Full model^f^**									
	**MI^g^ components**								
		Percentage change talk	−0.11	−0.20 to −0.01	.02^e^	−0.17		−0.32 to −0.02	.03^e^
		Any pros or cons listed	−1.17	−8.02 to 5.68	.74	0.34		−13.07 to 13.76	.96
		Number of pros	1.31	−0.05 to 2.67	.06	1.60		−0.46 to 3.67	.13
		Number of cons	−0.17	−0.83 to 0.50	.62	−0,14		−1.16 to 0.88	.78
		What is important	−0.36	−6.64 to 5.91	.91	5.52		−4.23 to 15.27	.27
	**CBT^h ^components**								
		Setting a start date	−1.41	−7.59 to 4.78	.65	17.50		−4.81 to 39.82	.12
		Setting a drinking goal	−2.15	−8.39 to 4.09	.50	2.88		−16.05 to 21.81	.77
		Completing another part of the moderation plan	−3.22	−9.38 to 2.94	.30	−13.32		−36.97 to 10.34	.27
		Noting alcohol use before DYD^i^	−4.40	−9.83 to 1.03	.11	−5.00		−11.53 to 1.53	.13
		Any high-risk situations	−8.31	−14.88 to −1.74	.01^e^	−11.86		−24.22 to 0.49	.06
		Number of high-risk situations	−1.82	−3.12 to −0.52	.01^e^	−2.15		−3.92 to −0.38	.02^e^
		Any strategies	−1.21	−8.22 to 5.80	.73	5.51		−5.08 to 16.58	.30
		Number of strategies	0.01	−0.47 to 0.49	.01^e^	0.64		−0.09 to 1.37	.09
		Exploring cravings	2.90	−11.19 to 16.98	.69	3.71		−11.74 to 19.17	.64
		Exploring relapse prevention	−1.65	−14.71 to 11.41	.80	−0.24		−15.42 to 14.95	.98
		Making a relapse plan	3.52	−14.92 to 21.97	.71	5.05		−15.03 to 25.13	.62
		Exploring thoughts about drinking	−0.41	−9.16 to 8.34	.93	−4.00		−15.09 to 7.09	.48
		Any monitoring of drinking	2.66	−3.00 to 8.32	.36	1.77		−4.38 to 7.93	.57
		Number of times drinking was monitored	0.00	−0.05 to 0.06	.87	−0.01		−0.07 to 0.04	.62

^a^Unadjusted coefficients are based on a series of models in which alcohol use at 3-month follow-up is regressed based on baseline alcohol use, covariates, and each single intervention component.

^b^Adjusted coefficients are based on a model in which alcohol use at 3-month follow-up is regressed based on baseline alcohol use, covariates, and all intervention components.

^c^For the null model only containing the covariates, the adjusted R^2^ value is 0.36.

^d^N/A: not applicable.

^e^*P* value <.05.

^f^For the full model, the adjusted R^2^ value is 0.38.

^g^MI: motivational interviewing.

^h^CBT: cognitive behavioral therapy.

^i^DYD: Down Your Drink.

### Sensitivity Analyses

When comparing the estimates from the linear model without log transformation (Table 3) with the model estimates including log transformations of alcohol use variables (adjusted R^2^ 0.20, see Table S2 in Multimedia Appendix 2), and the model estimates resulting from a negative binomial regression (McFadden R^2^ 0.31, see Table S3 in Multimedia Appendix 3), a higher number of listed high-risk situations predicted lower alcohol use at 3-month follow-up in both the model with log-transformation (B_adj_ −0.10, 95% CI −0.16 to −0.04, *P*=.001) and the negative binomial model (B_adj_ −0.07, 95% CI −0.12 to −0.03, *P*=.001). However, a higher number of listed strategies also predicted higher alcohol use at 3-month follow-up in both the log-transformed model (B_adj_ 0.04, 95% CI 0.01 to 0.06, *P*=.002) and the negative binomial model (B_adj_ 0.03, 95% CI 0.01 to 0.05, *P*=.01). No evidence was found in either of the sensitivity analyses for the effect of the percentage of change talk (log-transformed: B_adj_ 0.00, 95% CI −0.01 to 0.00, *P*=.07; negative binomial: B_adj_ 0.00, 95% CI –0.01 to 0.00, *P*=.22). The findings for these two components are therefore inconsistent. For all other components, none of the models showed an effect.

## Discussion

### Principal Findings

Participants were found to actively use both the MI and CBT components of the DYD website, with MI components used by more participants. The CBT components pertaining to relapse prevention and exploration of cravings were rarely used by participants. Change talk and sustain talk were elicited by the most participants (341/410, 83.2%, and 329/410, 80.2%, respectively); generally, more instances of change talk (median 8) than sustain talk (median 3) were reported, although the between-person variance was large (SD 10.02 and 2.43, respectively). One explanation for the more frequent use of MI components than of CBT components is that the former are presented at the beginning of the program. Participants were free to choose how to move through the program; however, the ordering may still have contributed to the more frequent use of the MI components. A significant finding was that a higher number of listed high-risk situations robustly predicted a greater reduction in alcohol use at 3-month follow-up (*P*=.02).

Analyses were repeated with log-transformed measures of alcohol use at baseline and 3-month follow-up and by applying negative binomial regression. All the models showed an effect of the number of listed high-risk situations; however, there were also some inconsistent findings. There were differences between the models in the effects of the percentage of change talk and the number of listed strategies to deal with high-risk situations. The number of strategies to deal with high-risk situations predicted higher alcohol use in the log-transformed and negative binomial models but not in the linear model without log transformation. The percentage of change talk was only found to have an effect on reduction of alcohol use in the linear model without log transformation. All other components showed null findings.

The lack of effects of the percentage of change talk found in the sensitivity models may be due to a lack of evidence rather than the absence of a true effect. A meta-analysis by Magill [10] summarized 58 MI process studies, including 21 on face-to-face alcohol interventions. A higher proportion of change talk was found to be related to the reduction of all risky behaviors, including reduction of alcohol use. It is noteworthy that the latest guidance on MI practice suggests removing the pros and cons decisional balance exercise [9], as it can have the undesired effect of encouraging sustain talk [23].

A higher number of noted strategies was associated with a slight increase in alcohol use at 3-month follow-up according to the sensitivity analyses. A possible reason for this seemingly paradoxical finding is that participants who are having more severe alcohol problems may work more extensively on the program. For this group, a web-based alcohol intervention may offer insufficient support to actually reduce their alcohol use, thereby possibly leading to increased alcohol use. As only some of the participants were asked to fill in the complete AUDIT questionnaire in the original trial, this hypothesis could not be tested in this study. However, a recent individual patient data meta-analysis (19 trials) revealed no difference in the effectiveness of internet interventions between binge drinkers and non–binge drinkers, nor any difference in effectiveness related to the amount of alcohol consumption at baseline (heavy drinkers vs nonheavy drinkers) [5].

Another component that is often considered an “active ingredient” of brief alcohol interventions is self-monitoring of alcohol use [24]. Self-monitoring (ie, entry of drinks into the Drinking Episodes Diary), was actively used at least once by only 141/410 (34.4%) of the active participants. Also, for participants who did use it, the amount of times they reported their drinking was very skewed: half of them reported their drinking a maximum of 9 times (mean 34.8, SD 75.73). Among DYD users, there was also a lack of active engagement with relapse prevention exercises and exercises on craving. Craving was previously found to be an important predictor of relapse [25].

Future research should focus on testing the roles of components of interest in encouraging alcohol reduction using a preregistered analysis plan while considering the influence of the ordering of components on their use and subsequent effects.

### Strengths and Limitations

This study made use of a large sample of active users of a web-based alcohol intervention program. These users were e-help–seekers who were not currently enrolled in a treatment pathway. Uniquely, in this study, we were able to analyze a large number of free-text responses, thus obtaining insight into how well the participants understood the questions and whether key MI and CBT components were actively used. The free-text responses also enabled the assessment of key MI mechanisms of change talk and sustain talk, which may influence drinking outcomes [26]. Whether the program encouraged change talk or softened sustain talk (counter change talk) over time could not be assessed in this study. The presence of these key MI mechanisms and the use of the separate components were assessed independently by two coders with high interrater reliability. Change talk and sustain talk were coded using the CLEAR coding system [19], which was developed for coding of in-therapy client language. To account for the amount of total activity within the program as a possible confounder, the total number of words was included as a covariate within the model. These analyses were exploratory and were not preregistered; several sensitivity analyses are therefore presented. It is possible that significance of the effects of some components may be detected when using a larger sample, as we included many parameters without any effects in the full model. The generalizability of the study is limited because the sample only consisted of participants whose alcohol use at 3-month follow-up was known; this complete case analysis limits the generalizability of the results to nonresponders. Furthermore, the DYD intervention was closely modeled on the face-to-face MI/CBT approach used in therapeutic settings, and it required engagement and reflection across many different exercises. DYD was only accessible on a computer (ie, not compatible with smartphones, which were less prolific in 2007 when DYD was first launched). The current results are therefore only generalizable to similar extensive web-based MI/CBT interventions. More recent web-based interventions are responsive websites or apps, which tend to include a small number of “active” behavior change components that can be used easily and quickly, and are not intended to elicit change talk or sustain talk.

### Conclusions

A web-based alcohol intervention was able to elicit both change talk and sustain talk. A higher number of listed high-risk situations can predict lower alcohol use at 3-month follow-up. Other components show inconsistent findings and should be studied further using a preregistered analysis plan. This study points to components of the DYD website that may constitute effective internet alcohol moderation programs and thus complies with the high research priority of studying specific components of web-based interventions.

## Appendix

Multimedia Appendix 1 Coding scheme.

Multimedia Appendix 2 Supplementary Table S2: Linear model estimates with log-transformed alcohol use variables at baseline (independent variable) and 3-month follow-up (dependent variable) for the active use sample (n=410).

Multimedia Appendix 3 Negative binomial model estimates for the active use sample.

## Abbreviations

AUDIT-C: Alcohol Use Disorders Identification Test-Consumption
CBT: cognitive behavioral therapy
CLEAR: Client Language EAsy Rating coding system
CTS-R: Revised Cognitive Therapy Scale
DYD: Down Your Drink
e-help: electronic help
GCSE: General Certificate of Secondary Education
MI: motivational interviewing
