# Supplementary material for: Engagement With Motivational Interviewing and Cognitive Behavioral Therapy Components of a Web-Based Alcohol Intervention, Elicitation of Change Talk and Sustain Talk, and Impact on Drinking Outcomes: Secondary Data Analysis
Source: J Med Internet Res. 2020 Sep 1;22(9):e17285. doi: 10.2196/17285 (PMC7492976; doi:10.2196/17285)
Supplement: Multimedia Appendix 1 [file jmir_v22i9e17285_app1.doc]

*Appendix S1
Coding scheme for responses within the Down Your Drink (DYD) programme*

| Code | Label | Coding criteria |
| --- | --- | --- |
| **CBT components** | | |
| **CBT1** | Has set a start date | Set a start date for working on drinking goal = 1  No response = 0 |
| **CBT2** | Has set a goal | Answered a question about goal setting with a drinking goal, either specific or unspecific = 1  Answered to questions about goal setting with a goal, but not related to alcohol use or no response = 0 |
| **CBT3** | Has completed another part of the moderation plan | Answered a question in the Making a Plan section other than goal setting or start date e.g. reviewing day, method, difficulties = 1  No response = 0 |
| **CBT4** | Has noted alcohol use prior to starting DYD | Filled in how many units they drank before starting DYD programme = 1  No response = 0 |
| **CBT5** | Has noted risky situations | Answered a question about potential risky situations that lead to drinking (more than intended) across multiple sections = 1  No response or not related to drinking = 0 |
| **CBT5.number** |  | Number of listed risky situations for drinking (more than intended) |
| **CBT6** | Has noted strategies to deal with risky situations | Answered a question about dealing with risky situations across multiple sections = 1  Not responded to questions about dealing with risky situations with meaningful strategies = 0 |
| **CBT6.number** |  | Frequency of listed strategies to deal with risky situations |
| **CBT7** | Has explored feelings of craving | Answered a question about cravings across multiple sections = 1  No response = 0 |
| **CBT8** | Has explored relapse prevention | Answered a question about relapse prevention across multiple sections = 1  No response = 0 |
| **CBT9** | Has made a relapse plan | Answered a question about a relapse plan e.g. who to call = 1  No response = 0 |
| **CBT10** | Has examined his/her thoughts about dirnking | Answered a question about his/her thoughts about drinking in ‘The Scottish Social Attitudes Survey (2004)’ or related sections = 1  No response = 0 |
| **CBT11** | Has monitored units of drinking | Monitored alcohol use at least once in Drinking Episodes Diary = 1  No response in the Drinking Episodes Diary = 0 |
| **CBT11.number** |  | Frequency of monitoring of drinking |
| **MI components** | | |
| **MI1** | Presence of change talk | Presence of change talk in free text responses across all parts of the programme= 1  No presence of change talk = 0 |
| **MI1.number** |  | Frequency of change talk statements |
| **MI2** | Presence of sustain talk | Presence of sustain talk in in free text responses across all parts of the programme = 1  No presence of sustain talk = 0 |
| **MI2.number** |  | Frequency of sustain talk statements |
| **MI3** | Has noted pros and cons of drinking | Answered a question about pros and cons of drinking across multiple sections = 1  No response or not related to drinking= 0 |
| **MI3.pronumber** |  | Number of listed pros of drinking |
| **MI3.connumber** |  | Number of listed cons of drinking |
| **MI4** | Has noted what is most important and meaningful to oneself | Answered a question about what really matters to them in their life = 1  No response = 0 |
| **General components** | | |
| **G1** | Has responded to any of the questions | Responded to at least one of the questions = 1  No response entered into the programme = 0 |

Engagement with the DYD programme corresponded to certain cognitive behavioural therapy (CBT) and motivational interviewing (MI) components which were coded in three ways. Firstly, they were coded in a binary fashion with presence (1) or no presence of response (0). Some components allowed for free text response in which each utterance/statement counted to provide frequencies. Lastly, change and sustain talk was recorded throughout responses using the CLEAR guidelines.

Coders ensured a clear connection to drinking was present in order to count it as change or sustain talk. Otherwise it was classified as neutral talk. We coded utterances, not number of unique risky situations or unique strategies to handle them etc. So repeatedly stating the same thing was also counted.

Example:

Utterance: “to drink less and be less dependent on it”

Coded:

- CBT2 (Set a goal) = 1
- MI1 (Presence of change talk) = 1
- MI1.number: counted as instance of change talk
