# Supplementary material for: Engagement With Motivational Interviewing and Cognitive Behavioral Therapy Components of a Web-Based Alcohol Intervention, Elicitation of Change Talk and Sustain Talk, and Impact on Drinking Outcomes: Secondary Data Analysis
Source: J Med Internet Res. 2020 Sep 1;22(9):e17285. doi: 10.2196/17285 (PMC7492976; doi:10.2196/17285)
Supplement: Multimedia Appendix 2 [file jmir_v22i9e17285_app2.doc]

*Supplementary table S2* *Linear model estimates with log-transformed alcohol use variables at baseline (independent variable) and 3-month follow-up (dependent variable) for the active use sample (n=410*)

| **Variables** | | **Active use sample (n=410)** | | | | | |
| --- | --- | --- | --- | --- | --- | --- | --- |
|  |  | Unadjusted^a^ | | | Adjusted^b^ | | |
|  |  | B | 95%CI | *P* value | B | 95%CI | *P* value |
| **Covariates** | | | | | | | |
|  | Baseline alcohol use | - | - | - | 0.63 | 0.49 to 0.78 | <.001 |
|  | Gender (male) | - | - | - | −0.05 | −0.23 to 0.13 | .59 |
|  | Education (A level) | - | - | - | 0.14 | −0.09 to 0.38 | .24 |
|  | Education (O level) | - | - | - | 0.13 | −0.13 to 0.38 | .32 |
|  | Education (other) | - | - | - | −0.07 | −0.40 to 0.25 | .65 |
|  | Education (no qualification) | - | - | - | −0.28 | −0.83 to 0.26 | .30 |
|  | Age | - | - | - | 0.01 | 0.00 to 0.02 | .04 |
|  | Number of words | - | - | - | 0.00 | 0.00 to 0.00 | .63 |
| **MI components** | | | | | | | |
|  | Percentage change talk | 0.00 | −0.01 to 0.00 | .02^d^ | 0.00 | −0.01 to 0.00 | .07 |
|  | Any pros or cons listed | −0.08 | −0.31 to 0.15 | .51 | −0.08 | −0.54 to 0.37 | .72 |
|  | Number of pros | 0.04 | 0.00 to 0.09 | .07 | .06 | −0.01 to 0.13 | .09 |
|  | Number of cons | −0.01 | −0.03 to 0.02 | .52 | −0.01 | −0.04 to 0.03 | .69 |
|  | What is important | −0.04 | −0.25 to 0.18 | .74 | 0.15 | −0.18 to 0.48 | .36 |
| **CBT components** | | | | | | | |
|  | Setting start date | −0.07 | −0.27 to 0.14 | .54 | 0.35 | −0.40 to 1.11 | .36 |
|  | Setting a drinking goal | −0.08 | −0.30 to 0.13 | .44 | −0.03 | −0.67 to 0.61 | .93 |
|  | Completing another part of moderation plan | −0.10 | −0.30 to 0.11 | .37 | −0.14 | −0.95 to 0.66 | .73 |
|  | Noting alcohol use before DYD | −0.11 | −0.29 to 0.08 | .26 | −0.12 | −0.34 to 0.10 | .29 |
|  | Any risky situations | −0.21 | −0.44 to 0.01 | .06 | −0.12 | −0.54 to 0.30 | .59 |
|  | Number of high-risk situations | −0.06 | −0.11 to −0.02 | .01^d^ | −0.10 | −0.16 −0.04 | .001^d^ |
|  | Any strategies | −0.13 | −0.37 to 0.11 | .29 | −0.14 | −0.51 to 0.22 | 0.44 |
|  | Number of strategies | 0.00 | −0.01 to 0.02 | .56 | 0.04 | 0.01 to 0.06 | 0.002^d^ |
|  | Exploring cravings | −0.03 | −0.50 to 0.45 | .91 | −0.07 | −0.60 to 0.45 | .79 |
|  | Exploring relapse prevention | 0.07 | −0.38 to 0.51 | .76 | 0.31 | −0.21 to 0.82 | .24 |
|  | Making a relapse plan | 0.04 | −0.59 to 0.67 | .90 | 0.09 | −0.59 to 0.77 | .80 |
|  | Exploring thoughts about drinking | −0.05 | −0.34 to 0.25 | .75 | −0.25 | −0.63 to 0.12 | .19 |
|  | Any monitoring of drinking | 0.12 | −0.07 to 0.31 | .22 | 0.11 | −0.09 to 0.32 | .28 |
|  | Number of times drinking was monitored | 0.00 | 0.00 to 0.00 | .99 | 0.00 | 0.00 to 0.00 | .38 |

*Note*. Null model only containing covariates adjusted R^2^ = 0.17, full model adjusted R^2^ = 0.20. ^a^ Unadjusted coefficients are based upon a series of models in which log-transformed alcohol use at three months follow-up is regressed upon baseline log-transformed alcohol use, covariates and each single intervention component. ^b^ Adjusted coefficients are based upon a model in which log-transformed alcohol use at three months follow-up is regressed upon baseline log-transformed alcohol use, covariates and all intervention components. ^c^ For interpretation of coefficients: exponentiated coefficients correspond with percentage increase of alcohol use after one unit increase of the independent variable. For interpretation of baseline alcohol use coefficient: every 10% increase in baseline alcohol use results in (1.10)^B^ increase in alcohol use at three month follow-up. ^d^ *P* value < .05.
