# Supplementary material for: Engagement With Motivational Interviewing and Cognitive Behavioral Therapy Components of a Web-Based Alcohol Intervention, Elicitation of Change Talk and Sustain Talk, and Impact on Drinking Outcomes: Secondary Data Analysis
Source: J Med Internet Res. 2020 Sep 1;22(9):e17285. doi: 10.2196/17285 (PMC7492976; doi:10.2196/17285)
Supplement: Multimedia Appendix 3 [file jmir_v22i9e17285_app3.docx]

Supplementary table S3
*Negative binomial model estimates for the active use sample (n=410)*

| **Variables** | | **Active use sample (n=410)** | | | | | |
| --- | --- | --- | --- | --- | --- | --- | --- |
|  |  | Unadjusted^a^ | | | Adjusted^b^ | | |
|  |  | B | 95%CI | *P* value | B | 95%CI | *P* value |
| **Covariates** | | | | | | | |
|  | Baseline alcohol use | - | - | - | 0.01 | 0.01 to 0.01 | <.001^c^ |
|  | Gender (male) | - | - | - | −0.08 | −0.22 to 0.07 | .29 |
|  | Education (A level) | - | - | - | 0.08 | −0.11 to 0.27 | .41 |
|  | Education (O level) | - | - | - | 0.12 | −0.07 to 0.33 | .22 |
|  | Education (other) | - | - | - | −0.07 | −0.32 to 0.19 | .58 |
|  | Education (no qualification) | - | - | - | −0.27 | −0.68 to 0.18 | .22 |
|  | Age | - | - | - | 0.00 | 0.00 to 0.01 | .04^c^ |
|  | Number of words | - | - | - | 0.00 | 0.00 to 0.00 | .41 |
| **MI components** | | | | | | | |
|  | Percentage change talk | 0.00 | 0.00 to 0.00 | .16 | 0.00 | −0.01 to 0.00 | .22 |
|  | Any pros or cons listed | −0.01 | −0.20 to 0.17 | .89 | −0.10 | −0.46 to 0.25 | .58 |
|  | Number of pros | 0.03 | 0.00 to 0.07 | .06 | 0.05 | −0.01 to 0.11 | .08 |
|  | Number of cons | 0.00 | −0.02 to 0.02 | .99 | 0.00 | −0.03 to 0.03 | .85 |
|  | What is important | 0.01 | −0.16 to 0.18 | .89 | 0.10 | −0.17 to 0.36 | .45 |
| **CBT components** | | | | | | | |
|  | Setting start date | −0.08 | −0.24 to 0.09 | .36 | 0.37 | −0.25 to 0.94 | .22 |
|  | Setting a drinking goal | −0.10 | −0.27 to 0.07 | .23 | −0.07 | −0.60 to 0.41 | .77 |
|  | Completing another part of moderation plan | −0.11 | −0.27 to 0.06 | .20 | −0.23 | −0.82 to 0.40 | .47 |
|  | Noting alcohol use before DYD | −0.06 | −0.21 to 0.08 | .41 | −0.07 | −0.24 to 0.10 | .44 |
|  | Any risky situations | −0.21 | −0.38 to −0.03 | .02^c^ | −0.17 | −0.51 to 0.15 | .30 |
|  | Number of high-risk situations | −0.05 | −0.09 to −0.02 | .002^c^ | −0.08 | −0.12 to −0.03 | .001^c^ |
|  | Any strategies | −0.08 | −0.27 to 0.11 | .40 | 0.03 | −0.26 to 0.32 | .83 |
|  | Number of strategies | 0.00 | −0.01 to 0.01 | .77 | .03 | 0.01 to 0.05 | <.001^c^ |
|  | Exploring cravings | −0.16 | −0.53 to 0.23 | .40 | −0.15 | −0.55 to 0.27 | .47 |
|  | Exploring relapse prevention | −0.13 | −0.48 to 0.24 | .48 | 0.14 | −0.28 to 0.57 | .51 |
|  | Making a relapse plan | −0.02 | −0.48 to 0.49 | .94 | 0.08 | −0.44 to 0.63 | .78 |
|  | Exploring thoughts about drinking | −0.08 | −0.31 to 0.16 | .51 | −0.23 | −0.53 to 0.08 | .13 |
|  | Any monitoring of drinking | 0.07 | −0.08 to 0.23 | .34 | 0.10 | −0.06 to 0.26 | .23 |
|  | Number of times drinking was monitored | 0.00 | 0.00 to 0.00 | .72 | 0.00 | 0.00 to 0.00 | .24 |

*Note*. Null model only containing covariates McFaddens’ R^2^ = 0.26, full model McFaddens’ R^2^ = 0.31. ^a^ Unadjusted coefficients are based upon a series of models in which alcohol use at three months follow-up is regressed upon baseline alcohol use, covariates and each single intervention component. ^b^ Adjusted coefficients are based upon a model in which alcohol use at three months follow-up is regressed upon baseline alcohol use, covariates and all intervention components. ^c^ *P*-value < .05.
